# Supplementary material for: Modeling glioblastoma heterogeneity as a dynamic network of cell states
Source: Mol Syst Biol. 2021 Sep 16;17(9):e10105. doi: 10.15252/msb.202010105 (PMC8444284; doi:10.15252/msb.202010105)
Supplement: Supplementary file 6 — Source Data for Figure 5 [file MSB-17-e10105-s004.zip › Figure5A_sourcedata/GSEA_3017/nosigdb_stateF.GseaPreranked.1622795015644/DOROSZKO_U3180MG_CLUSTER_5.html]

Details for gene set DOROSZKO\_U3180MG\_CLUSTER\_5[GSEA]

|  || Dataset | state33017 |
| Phenotype | NoPhenotypeAvailable |
| Upregulated in class | na\_pos |
| GeneSet | DOROSZKO\_U3180MG\_CLUSTER\_5 |
| Enrichment Score (ES) | 0.68770164 |
| Normalized Enrichment Score (NES) | 2.8294659 |
| Nominal p-value | 0.0 |
| FDR q-value | 0.0 |
| FWER p-Value | 0.0 |
Table: GSEA Results Summary

  

Fig 1: Enrichment plot: DOROSZKO\_U3180MG\_CLUSTER\_5      
 Profile of the Running ES Score & Positions of GeneSet Members on the Rank Ordered List

  

| PROBE | GENE SYMBOL | GENE\_TITLE | RANK IN GENE LIST | RANK METRIC SCORE | RUNNING ES | CORE ENRICHMENT || 1 | CCL2 |  |  | 7 | 1.774 | 0.1122 | Yes |
| 2 | LGALS3 |  |  | 16 | 1.261 | 0.1905 | Yes |
| 3 | SLC4A4 |  |  | 40 | 0.980 | 0.2438 | Yes |
| 4 | GFAP |  |  | 47 | 0.953 | 0.3030 | Yes |
| 5 | CXCL14 |  |  | 51 | 0.931 | 0.3622 | Yes |
| 6 | LPL |  |  | 59 | 0.874 | 0.4158 | Yes |
| 7 | AQP4 |  |  | 84 | 0.789 | 0.4563 | Yes |
| 8 | CRYAB |  |  | 85 | 0.783 | 0.5072 | Yes |
| 9 | NRN1 |  |  | 107 | 0.741 | 0.5459 | Yes |
| 10 | NCAN |  |  | 138 | 0.686 | 0.5771 | Yes |
| 11 | NAMPT |  |  | 168 | 0.634 | 0.6053 | Yes |
| 12 | CD44 |  |  | 191 | 0.600 | 0.6344 | Yes |
| 13 | PLA2G16 |  |  | 284 | 0.523 | 0.6271 | Yes |
| 14 | POSTN |  |  | 296 | 0.511 | 0.6554 | Yes |
| 15 | CYSTM1 |  |  | 358 | 0.484 | 0.6595 | Yes |
| 16 | ID2 |  |  | 366 | 0.483 | 0.6877 | Yes |
| 17 | GJA1 |  |  | 495 | 0.436 | 0.6587 | No |
| 18 | S100A11 |  |  | 692 | 0.373 | 0.5951 | No |
| 19 | FAM107A |  |  | 718 | 0.367 | 0.6077 | No |
| 20 | ID3 |  |  | 789 | 0.349 | 0.5990 | No |
| 21 | PDLIM4 |  |  | 982 | 0.313 | 0.5333 | No |
| 22 | TRIM47 |  |  | 1353 | 0.262 | 0.3844 | No |
| 23 | EMP1 |  |  | 1598 | -0.285 | 0.2936 | No |
Table: GSEA details [plain text format]

  

Fig 2: DOROSZKO\_U3180MG\_CLUSTER\_5: Random ES distribution      
 Gene set null distribution of ES for **DOROSZKO\_U3180MG\_CLUSTER\_5**
